# Rolling Circles as a Means of Encoding Genes in the RNA World

**DOI:** 10.3390/life12091373

**Published:** 2022-09-02

**Authors:** Felipe Rivera-Madrinan, Katherine Di Iorio, Paul G. Higgs

**Affiliations:** Department of Physics and Astronomy, Origins Institute, McMaster University, Hamilton, ON L8S 4L8, Canada

**Keywords:** rolling circle, RNA world, computer simulation, error threshold

## Abstract

The rolling circle mechanism found in viroids and some RNA viruses is a likely way that replication could have begun in the RNA World. Here, we consider simulations of populations of protocells, each containing multiple copies of rolling circle RNAs that can replicate non-enzymatically. The mechanism requires the presence of short self-cleaving ribozymes such as hammerheads, which can cleave and re-circularize RNA strands. A rolling circle must encode a hammerhead and the complement of a hammerhead, so that both plus and minus strands can cleave. Thus, the minimal functional length is twice the length of the hammerhead sequence. Selection for speed of replication will tend to reduce circles to this minimum length. However, if sequence errors occur when copying the hammerhead sequence, this prevents cleavage at one point, but still allows cleavage on the next passage around the rolling circle. Thus, there is a natural doubling mechanism that creates strands that are multiple times the length of the minimal sequence. This can provide space for the origin of new genes with beneficial functions. We show that if a beneficial gene appears in this new space, the longer sequence with the beneficial function can be selected, even though it replicates more slowly. This provides a route for the evolution of longer circles encoding multiple genes.

## 1. Introduction

It is likely that RNA replication arose very early in the history of life and that there was an RNA World stage at which RNA molecules carried out the roles of both genes and catalysts [1,2]. There has been considerable progress with development of RNA polymerase ribozymes in the laboratory [3,4,5,6], which makes the existence of such ribozymes in the RNA World seem plausible, although there is still no ribozyme that is able to repeatedly copy its own sequence. Furthermore, as a newly evolved polymerase would require a complementary minus strand on which to act, it seems likely that some form of non-enzymatic replication would be required prior to the origin of polymerases in order to generate the complementary strand at the same time as the polymerase. In recent years, there has been considerable experimental progress on non-enzymatic replication [7,8,9,10,11,12], although important details are still not understood.

Non-enzymatic synthesis of a complementary strand on a template leads to formation of a double strand. Double strands are stable under conditions in which the templating reaction occurs. This suggests that a temperature cycling process is required to separate the double strands and allow further cycles of replication. However, on cooling, reannealing of existing strands is rapid compared to synthesis of new complementary strands, therefore replication driven by temperature cycling is blocked by reannealing. In a very simple model dealing with replication of a single type of plus and minus strand, reannealing inhibits replication at a very low strand concentration [13]. However, the possibility remains that in a diverse mixture of random sequences, reannealing of partially matching strands can lead to configurations in which productive primer extension and ligation can continue to occur [14]. It is known that functional ribozymes can be assembled by ligation in sequence mixtures that contain all the required fragments of the ribozymes [15,16], and it was suggested [14] that sequence information could be encoded in a mixture if the fragments formed a virtual circular genome. However, recent computer simulations of non-enzymatic synthesis in mixtures of RNA fragments [17] show that functional sequences become scrambled, even in the limit of zero mutational error, and that sequence information cannot be passed on.

In view of the problems above, we have suggested that the rolling circle mechanism is a more likely way to get replication started in the RNA World [13]. Rolling circle replication does not require temperature-cycling to melt double strands because the old complementary strand is gradually displaced from the template at the same time the new strand is synthesized. In rolling circle replication, multiple copies of a complementary strand are synthesized by repeatedly going around the same template strand. The growing strand contains a self-cleaving hammerhead ribozyme (HHRz) which cleaves the tail of the growing strand at a set position within the template. The linear strands produced by the cleavage have the ability to re-circularlize and reinitiate rolling circle replication. The smallest replicating RNAs in modern biology are viroids that use the rolling circle mechanism [18]. The idea that circular RNAs may be relics of the RNA World has been suggested some time ago [19,20]. Though viroid replication depends on protein polymerases, ribozyme-driven replication on circular templates has also been shown in the laboratory [6,21]. Non-enzymatic strand displacement has also been shown to some extent in the laboratory, but is very slow [22]. If non-enzymatic replication via the rolling circle mechanism can be achieved, as we proposed [13], this would be a likely point of origin of RNA replication, because the geometry and chemical nature of the reaction occurring at the point of RNA synthesis would remain the same through the initial non-enzymatic stage, a ribozyme catalyzed stage, and eventually a protein catalyzed stage. Thus a smooth pathway of evolution would be possible. This scenario beginning with circular templates is supported by recent experiments [23,24] showing that circular strands can arise during random polymerization of nucleotides.

All RNA World scenarios require a supply of nucleotides. If we follow a “replication-first” argument, then we assume that there was an abiotic pathway that produced nucleotides by chemical means. If we follow a “metabolism-first” argument, then we say that there was a pre-existing small-molecule autocatalytic metabolism that was able to synthesize nucleotides. In what follows, we assume that RNA replication is happening inside protocells. We assume that a supply of nucleotides is available from abiotic synthesis outside the cells, but it would not make any difference to the results if we assumed nucleotides are synthesized by a metabolism inside the cells. RNA World scenarios also need to assume that functional sequences can arise within mixtures of random sequences. This seems reasonable, because it has been demonstrated that many types of functional ribozymes can be selected from random pools by in vitro selection methods. However, the shorter and simpler the ribozyme, the more easily it will arise in a random mixture. Many types of self-cleaving ribozymes are known [25,26,27,28,29,30,31], but we are most interested in the hammerhead ribozyme here because of its presence in naturally occurring viroids, and because it is short and simple. The hammerhead is thought to have arisen multiple times in evolution, and arises relatively easily from random RNA pools in in vitro selection experiments [32]. If circular strands could be replicated non-enzymatically, then the first kind of ribozyme that would be required in the history of life would be a hammerhead, which would be much easier to evolve than the more complicated polymerase ribozyme. 

In the current paper we assume that non-enzymatic replication of small circles is possible, and we ask whether ribozymes of some beneficial function can be encoded on such circles. We suppose that circles are contained in lipid vesicles (protocells) that can divide when the RNA strands are multiplying within them. Beneficial genes that increase the rate of RNA replication by some means will also increase the rate of cell growth and division and will hence be selected. Evolutionary protocell models show that genes with beneficial functions can be selected in protocells, whereas they would not be selected in a well-mixed system without compartments [33]. The beneficial function could be a polymerase ribozyme which increases the rate of replication above the non-enzymatic rate, or a nucleotide synthetase ribozyme that increases the availability of monomers, or a ribozyme involved in lipid synthesis that increases the availability of membrane lipids. Several computational models for the cooperation of genes of these types have been studied [34,35,36,37]. Strands encoding beneficial genes are bound to be longer than minimal-length strands that have no function other than their own replication. Hence, they will have a disadvantage of replicating more slowly inside a cell. In the rolling circle case that we consider here, the minimal length circle is double the length of the hammerhead ribozyme (as it is required to cleave on both plus and minus strands). We therefore consider competition between minimal-length circles and longer circles that encode additional beneficial genes as well as the hammerheads. 

This problem is related to the evolution of strands containing linked genes. It was shown some time ago [38] that RNA strands having two linked genes with different functions could out-compete two separate strands having only one gene each. The linked strand has the advantage that the two genes replicate and segregate together, but the disadvantage that replication of the longer strand is slower. 

The case of ribozymes on circular chromosomes was studied in [35], where it was argued that circular chromosomes have advantages over linear chromosomes because replication can start at any point in the sequence and because the ends of linear chromosomes tend to degrade. Our own reasons for favoring circular chromosomes are somewhat different. We have argued that temperature cycling mechanisms to separate double strands are not efficient, either because they are inhibited by reannealing [13], or they cannot replicate sequence information [17], and therefore, that a strand displacement mechanism is required that does not require temperature cycling. We also showed that strand displacement from a linear template is very difficult because the short growing complementary strand is repeatedly displaced by the pre-existing complete complementary strand before it can reach completion [13]. Replication on a circular template avoids this problem. Therefore, in the current paper, we assume that only circular strands can replicate. Linear strands which are cleaved from the rolling circle have some probability of re-circularizing and becoming templates and some probability of forming folded structures that cannot replicate, but which can be functional ribozymes if they contain the correct sequence.

Selection for rapid replication tends to reduce template length to the minimum, and therefore acts against encoding beneficial genes, as in previous models [34,35]. However, we show that this is countered by an inherent mechanism in rolling circles that leads to length doubling. Cleavage of the tail from a rolling circle can only occur if the sequence of the hammerhead ribozyme is correctly copied from the template. If a sequence error occurs during copying of this ribozyme, the tail grows longer until the same point is reached on the subsequent cycle. This time, if the sequence is correctly copied, a double-length strand is released, which can form a double-length circle. We firstly investigate the distribution of sequence lengths expected under the action of this doubling mechanism. We then discuss the requirements for a longer circle containing a beneficial gene to become fixed within a population.

## 2. Materials and Methods

### 2.1. Basic Mechanism of Rolling Circle Replication

We simulate a population of RNAs with differing lengths and sequences. Rather than store full RNA sequences at the single base level, we will represent sequences as strings of characters where each character represents an RNA section of length roughly 25 bases. The hammerhead ribozyme (HHRz) is represented as two characters **AZ**, where **A** and **Z** are the 5’ and 3’ parts of the ribozyme that are formed when it cleaves. A HHRz is typically 50 nucleotides long; hence our choice of 25 for the length represented by one character. We use the * character to indicate an RNA section with no particular sequence or function. Any sequence, such as ****AZ**, which contains the **AZ** motif can cleave between the **A** and the **Z**. The HHRz is able to re-ligate, leading to the formation of a new circular strand [29]. In our model, any sequence, such as Z*******A, with **Z** at its 5’ end and **A** at its 3′ end can circularize. Copying **A** and **Z** motifs creates their complementary sequences, denoted by **a** and **z**. Copying **a** and **z** recreates **A** and **Z**. Replication proceeds in the reverse direction (3′ to 5′ on the template), therefore the complement of **AZ** is **za**. We assume that **za** is not itself a HHRz, but it encodes a sequence that is complementary to the ribozyme. For repeated replication to occur, the sequence must contain both **AZ** and **za;** therefore, the minimal replicating circle has sequence **ZzaA**. 

**Figure 1:**
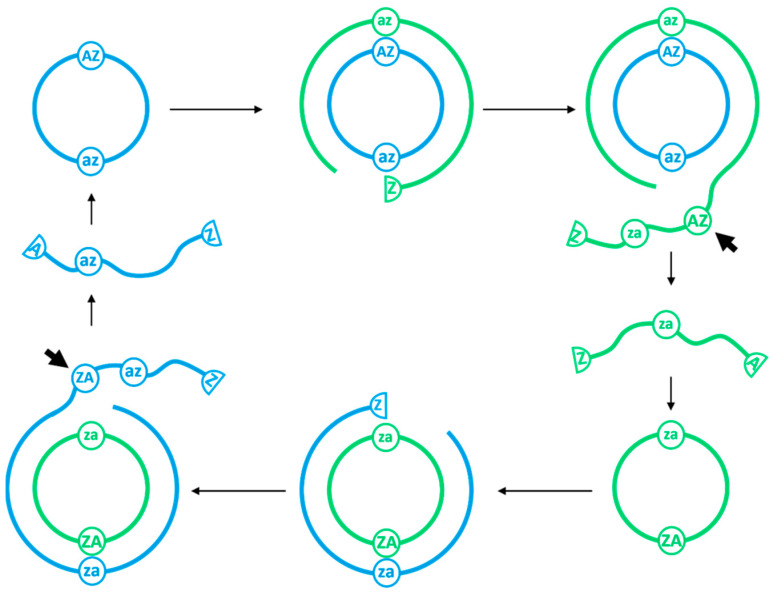
Mechanism of non-enzymatic rolling circle replication. Blue and green strands are complimentary plus and minus strands, each of which contains a ribozyme unit **AZ** and its complement **za**. After one circuit around the template, a double strand is created. After another circuit, a tail is produced. When the **AZ** motif is exposed in the tail, cleavage occurs, creating a linear strand that can circularize and begin the cycle anew.

If we begin copying the template at **z** and proceed in the reverse direction around the circular template, then we generate a complementary sequence **ZzaAZzaAZzaA....** which can cleave at each occurrence of the **AZ** motif to give multiple copies of **ZzaA**. This can re-circularize and continue replication. This is illustrated in Figure 1. The **ZzaA** sequence is self-complementary at the level of the character representation, but it need not be at the single nucleotide level. For example, there may be two HHRz sequences, **A_1_Z_1_** and **A_2_Z_2_** with equivalent function but different base sequences. In this case, there would be plus and minus strands **Z_1_z_2_a_2_A_1_** and **Z_2_z_1_a_1_A_2_**, illustrated by blue and green strands in Figure 1. Copying each of these gives rise to the other.

### 2.2. Model Details

Each RNA strand in the model is stored as a string of characters, as described above. A rate $r_{i}\;$ is assigned to each sequence *i* in the model, which is the rate of the next event that can happen to that sequence. Events are of different types but there is always only one possible next event for each sequence. The method of Gillespie [39] is used to simulate the stochastic occurrence of events. At each time step we calculate $R_{tot}=\underset{i}{\sum }r_{i}$, then a strand is chosen randomly with a probability $r_{i}/R_{tot}$. The event corresponding to sequence *i* then occurs. The time is updated by an amount δt, which is a random variable chosen from the probability distribution $P\left(\delta t\right)=R_{tot}\exp\left(-R_{tot}\delta t\right)$. Thus, exactly one random event occurs in every time step.

A strand may either be linear or circular. A linear strand is able to fold to a structure at rate $R_{fold};$ therefore we set the rate $r_{i}\;$ to be $R_{fold}$ for linear strands. For a linear strand that begins with **Z** and ends with **A**, the folding event brings the two ends together in the appropriate arrangement for ligation. When this event occurs, the linear strand becomes a single-stranded circle. A linear strand that does not have both **Z** and **A** at its ends cannot form a circle. When folding of such a strand occurs, it forms a folded linear strand that does not undergo any further events. In this case, we set $r_{i}$ = 0, so it is never selected for further events. 

The rate $r_{i}$ for a single stranded circle is set to $R_{syn}$, the rate of synthesis of a single character in the complementary strand. When this event occurs, a random position is chosen on the template circle and a complementary sequence is initiated with the character that is complementary to the template character. An example is shown in Table 1, Event 1. The sequence before the colon denotes the template and the sequence after the colon denotes the complementary strand. The variable *last* is used to store the position on the template of the last character added. In this case, the **A** that has been synthesized is complementary to the **a** in the template, which is at position 3 in the template; therefore *last* = 3.

When the complementary strand is shorter than the length of the template circle, we suppose that synthesis of the next complementary character occurs at the same rate $r_{i}=R_{syn}$. When this event occurs, an extra character is added to the complementary sequence, as in Event 2 in Table 1. As synthesis goes in the reverse direction on the template, *last* is decreased by 1. If *last* was 1 prior to the event, then it is set to the length of the template (because the template is circular). Synthesis can proceed by several events until the length of the complementary strand reaches the length of the template circle (Events 3 and 4).

At this point, further addition to the 3’ end of the growing strand requires displacement of the 5’ end. We set the rate to $r_{i}=R_{dis}$ in this case. We assume that the rate of synthesis involving strand-displacement, $R_{dis}$, is slower than the rate of synthesis on an unobstructed single strand, $R_{syn}$, as discussed by Tupper and Higgs [13]. The complementary strand produced after Event 5 has a single-stranded tail emerging from the circle. This is the initial **A** at the 5’ end of the complementary sequence (underlined in Table 1). After one further event, the tail has grown to **AZ**. We assume that the ribozyme cleaves instantaneously whenever the **AZ** motif arises in the free tail. In this case, a single **A** is cleaved off. This **A** is a linear strand which cannot circularize. It will fold to form a folded linear strand that plays no active role. The complementary strand on the circle can continue to grow by strand displacement. After several more steps, Event 7 will occur, which leads to cleavage of a complete **ZzaA**, which will circularize and initiate replication. Thus the cycle is complete.

The first sequence to be cleaved off is usually an incomplete fragment that cannot circularize, as with the single **A** in this case. The length of this fragment will depend on the position at which the first synthesis event occurs on a new circle. Only if the new strand is initiated with the **Z** will the first sequence formed be complete. However, once one fragment is released, subsequent sequences cleaved will always be complete (i.e., equal length to the template).

### 2.3. Mutations

In the above examples, we assumed perfectly accurate sequence replication, so that each character in the template always created its complementary character in the complementary strand. However, in the simulations below, we allow deleterious mutations to occur with probability *u* per character. Characters **A**, **a**, **Z**, and **z** represent specific sequences with structure and function. Each of these is accurately replicated to its complementary character with probability 1 − *u*. With probability *u*, a deleterious mutation occurs. The new character is *, which represents a sequence with no function. If the template character is *, this is always copied to another *. We ignore mutations that create functional characters from * because they are expected to be much rarer than deleterious mutations.

**Figure 2:**
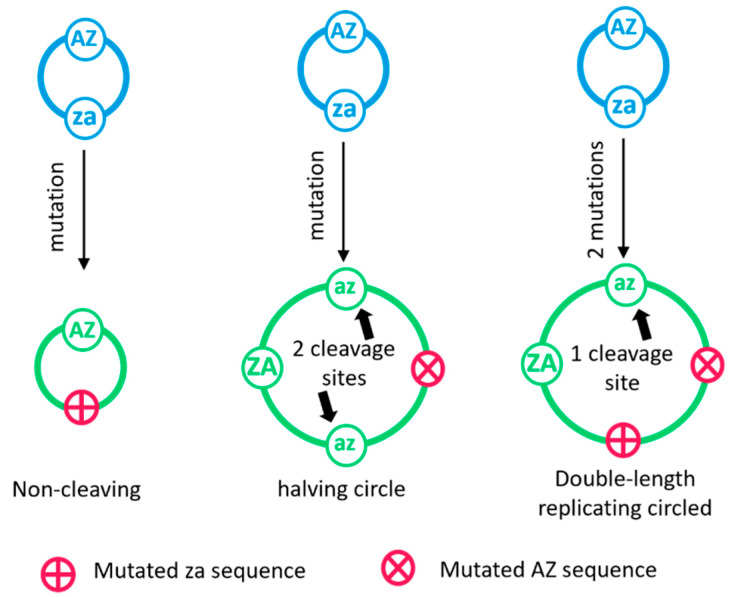
Mutations when copying minimal length circles give three kinds of strands with different behavior. A mutation in the **za** motif (either z*, *a, or **) gives a non-cleaving circle which produces a complementary strand that can never cleave. A mutation in the **AZ** motif creates a halving circle which goes on to produce circles of half its own length. A mutation in both motifs produces a circle of double the original length that can stably replicate.

Mutations in the hammerhead motifs can create strands that have interesting properties. Suppose we are copying the template **ZzaA**, and a mutation occurs when either the **z** or the **a** is being synthesized. The resulting sequence is a 4-mer Z*aA or Zz*A. Both of these sequences can circularize because they have intact **Z** and **A**. However, if replication begins on these templates, the complement will not contain the **AZ** motif, therefore the tail will never cleave. Thus if a mutation occurs in the **za** motif, this leads to circles whose tails can never cleave. We call these non-cleaving circles (as shown in Figure 2).

On the other hand, if a mutation occurs when the **AZ** motif is being synthesized, this produces ZzaA* or Zza*Z in the growing tail. These tails do not cleave at the appropriate point because they do not contain **AZ**, however if there is no mutation on the next time around the circle, then we arrive at a tail such as ZzaA*zaAZ, which cleaves to give ZzaA*zaA. This is a double-length strand (an 8-mer) that can circularize. It contains only one functional **AZ** motif but two **za** motifs. We call this a halving circle (see Figure 2), because if replication begins on this 8-mer, it will cleave in two places, producing two 4-mers. If no further mutations occur, replication of ZzaA*zaA will produce the reproducing 4-mer **ZzaA**, and the non-cleaving sequence Z*aA.

If a second mutation occurs during replication of the halving sequence, then one of the two cleaving motifs can disappear. This produces an 8-mer such as Zza*Z*aA, that can accurately replicate because it contains one **AZ** and one **za**. This sequence could also have been produced if two mutations occurred at once during replication of the initial 4-mer. The remaining half motifs in the 8-mer will soon disappear by further mutation because there is no longer any selection acting on them. This would yield Zza****A. We see that mutations in the hammerhead motifs give a built-in mechanism by which longer-length rolling circles can be created. All possible multiples of the original 4-mer can be made. For example, 12-mers can be made directly from a 4-mer if there are mutations on two passages around the circle but not on the third—16-mers can be made by doubling an 8-mer, and so on. A minimal length 4-mer encodes only the ribozyme motifs necessary for its own replication. However, if longer sequences arise containing non-functional * regions, it is possible for beneficial functional sequences to eventually arise in these regions by de novo mutation. This means that the initial 4-mers could evolve from simple minimal replicators to chromosomes that encode genes that are of useful function to the cell that contains them. We investigate the possibility of encoding beneficial genes further below, after first considering replication of circles without any other function.

### 2.4. Protocell Compartments

We consider a system in which circles are replicating inside protocell compartments. Monomers are supplied from outside. The protocell membranes are assumed to be permeable to monomers but not RNA strands. Cell division is coupled to replication of the rolling circles. When the number of strands in any one cell reaches a maximum $S_{0}$, the cell divides in two, with strands distributed randomly between the two daughter cells. The program has *N* memory slots defined for cells, which allows up to *N* cells to be present in the population. When a cell division occurs, one of the daughter cells remains in the memory slot of the parent, and the other one is placed in a random memory slot, overwriting whatever was in that slot. This insures that cells compete for limited resources. There is also a small loss rate $R_{loss}$ at which cells become empty at random. We include this loss rate in addition to maintaining the limit of *N* cells in order to ensure that cells that cannot replicate are destroyed eventually. This guarantees the existence of an error threshold, i.e., a maximum mutation rate above which the whole population of cells dies out because the average cell division rate becomes slower than $R_{loss}$.

Compartments are often introduced into models of RNA replicators as a means of preventing the invasion of parasitic sequences. In models for polymerase ribozymes, parasites will destroy the polymerases in a well-mixed system, but the polymerases survive when replication occurs in compartments [2,33,40]. Selection at the level of compartments selects for cooperatively replicating sequences which enable rapid cell growth and division. This can overcome selection at the level of individual sequences, which favors rapidly replicating parasites. In the current model, however, we are assuming that replication is non-enzymatic, and it does not depend on cooperative polymerase ribozymes. A population of rolling circles replicating non-enzymatically would survive even in a well-mixed system, if the mutation rate were not too high to prevent replication of the hammerhead ribozyme. However, we are also interested here in the possibility of selection for beneficial genes encoded on the rolling circles. These genes cannot survive in a well-mixed system because any benefit they create would be diluted across the whole system and would give a very small benefit to all sequences equally, whether or not they encoded the beneficial gene. If protocell compartments are present, then the effects of a beneficial gene apply only to sequences in the same cell. The presence of a beneficial gene therefore benefits the sequence that encodes the gene relative to sequences in other cells that do not possess the gene. As the existence of protocells is essential for the evolution of beneficial genes, we will introduce protocells into this model right from the beginning.

## 3. Results

### 3.1. Basic Model—Length Distributions and Error Threshold Behaviour

The model was run with parameters shown in Table 2. We used values reported in the literature to approximate the orders of magnitude between our parameters. The rate of non-enzymatic addition of a 25-base character with strand displacement, $R_{dis}$, was defined as our point of reference with a value of 1 h^−1^, and we assumed that the $R_{syn}$ rate (which does not require strand displacement) is 100 times faster. When probing for an error threshold, as we do later in this section, the rate of cell death $R_{loss}$ is defined as 1 × 10^−3^ h^−1^. This is much less than the rate of cell division when the mutation rate is low, but when u approaches the error threshold, cell division becomes very slow and eventually falls below $R_{loss}$. When *u* is small and we are not close to an error threshold, it makes little difference whether $R_{loss}$ is zero or has a small positive value. We expect folding of a strand into its secondary structure to be a relatively fast event, so we set $R_{fold}$ to 100 h^−1^. We do not know exact values for these rate parameters but we aim to correctly identify fast and slow steps. Though we use hours as our time unit for simplicity, it is only the relative size of the rates that effects the outcome of our model. 

**Figure 3:**
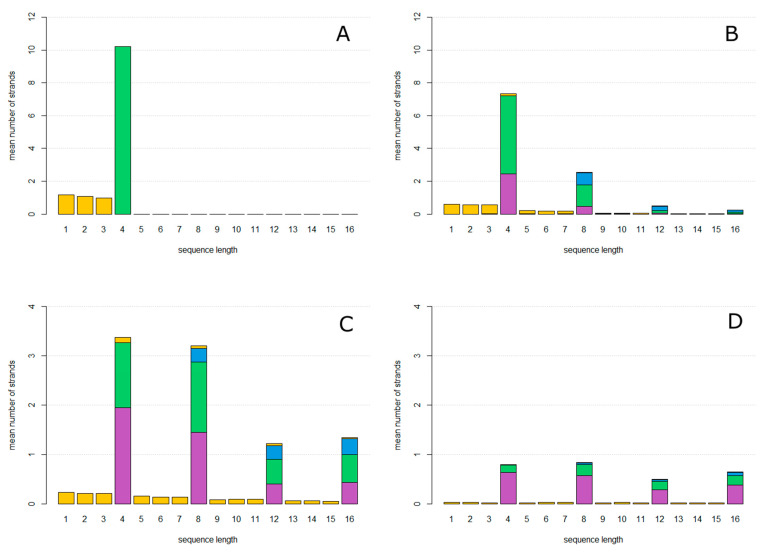
Mean number of strands of each length per cell. Colours indicate strand type: reproductive strands (green), non-cleaving strands (magenta), halving strands (blue), and non-circular fragments (yellow). (**A**), mutation probability *u* = 0. (**B**), mutation probability *u* = 0.15. (**C**), mutation probability *u* = 0.3. (**D**), mutation probability *u* = 0.45. $u_{indel}$ = 0 for all graphs.

We begin with a single circular **ZzaA** sequence per cell. After allowing the system to reach a steady state, the mean number of strands per cell of each length and type was counted and averaged over time. Strands are classified as one of four types, indicated by colours in Figure 3. Reproducing strands are those which have one complete **AZ** motif and one complete **za**. Non-Cleaving strands have one complete **AZ** motif and no **za**. Halving strands are those which have one complete **AZ** and more than one complete **za**. Fragments are those which do not have **Z** and **A** at the ends, and which therefore cannot circularize. 

In Figure 3A, the mutation probability is 0. The only sequences that arise are accurate copies of **ZzaA**, which are reproducing strands of length 4, and fragments of lengths less than 4 which are created as the first product strand of each new rolling circle. In Figure 3B, the mutation probability is *u* = 0.15. Circles of lengths equal to multiples of 4 are now seen, as well as fragments of all the other lengths. Non-Cleaving and Halving circles are found as well as Reproductive circles. 

**Figure 4:**
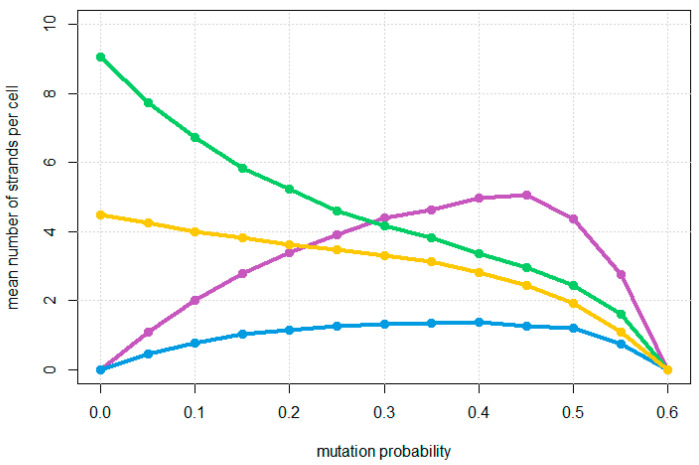
Mean number of strands per cell of the four types as a function of mutation probability *u*. Colours indicate reproductive strands (green), non-cleaving strands (magenta), halving strands (blue), and non-circular fragments (yellow). Strands of different lengths of each type are combined.

As the mutation probability increases as shown in Figure 3C (*u* = 0.3) and Figure 3D (*u* = 0.5), the number of reproductive circles decreases and the relative proportion halving and non-cleaving strands increases until the system meets an error threshold where replication can no longer keep up with the loss rate, as shown in Figure 4. At this point, all types of strands disappear, because they cannot be maintained without continued replication of the reproductive circles. The observed error threshold of *u* = 0.6 is the probability of a deleterious mutation in a single character (**A**, **Z** etc.). As each character represents a length roughly 25 bases, $u=25u_{per-base}f_{del}$, where $u_{per-base\;}$ is the point mutation rate per base, and $f_{del}$ is the fraction of mutations that are deleterious, i.e., the fraction of mutations within the structural region of the hammerhead that destroy the function of the hammerhead. This region contains loops whose sequence may not be important and paired regions in which compensatory changes may occur. Thus, $f_{del}$ may be considerably less than 1. If we assume $f_{del}=0.5$, then an error threshold of 0.6 in *u* corresponds to a threshold of 0.048 in $u_{per-base}$, which is not unreasonably small. The threshold value of 0.6 is dependent on the loss rate of cells, and would be higher if the loss rate were lower.

### 3.2. Insertions and Deletions

The 4-mer **ZzaA** is the shortest sequence having both the hammerhead and its complement. It is therefore the most rapidly replicating type of circle in this model. There is no reason why the self-cleaving ribozymes should initially appear on a minimal length sequence. However, we expect there to be strong selection for speed of replication; therefore if the hammerhead motifs initially appear on a longer circle, we expect relatively quick evolution toward the minimal-length circle due to deletions of the non-functional parts of the circle. Insertions and deletions (indels) can occur via the slipped-strand mispairing mechanism [41]. This tends to produce short tandem repeats which are often seen in viral genes [41,42]. 

Indels are introduced into the model in the following way. Each time a character is copied, an insertion occurs with probability $u_{indel}$, a deletion occurs with probability $u_{indel}$, or neither with probability $1-2u_{indel}$. In the case of an insertion, a * character is inserted in the growing sequence after the character that was just copied. In the case of a deletion, the character that was just copied is deleted, but the position of growing end on the template (indicated by *last*) still advances by one. 

**Figure 5:**
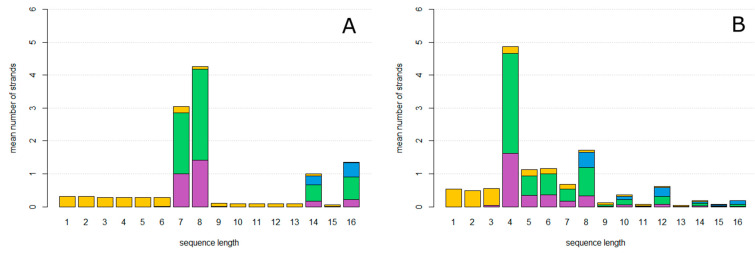
Mean number of strands of each length per cell, beginning from a single 8-mer per cell, including point mutations with probability *u* = 0.15 and indels with probability *u_indel_* = 0.015. Colours indicate reproductive strands (green), non-cleaving strands (magenta), halving strands (blue), and non-circular fragments (yellow). (**A**) Simulation steps elapsed T = 2 × 10^4^ (**B**) Simulation steps elapsed T = 2 × 10^6^.

Figure 5 considers a case where the first replicating circle that arises happens to be an 8-mer Zza****A. Each cell in the population begins with one copy of this 8-mer. The mutation rate is *u* = 0.15 and the indel rate is *u_indel_* = 0.015. After a relatively number of time steps in the Gillespie algorithm T = 2 × 10^4^, shown in Figure 5A, 7-mers have evolved from this by deletion, and double-length circles have arisen from both the 8-mer and the 7-mer by the doubling mechanism described above. After a much longer number of steps T = 2 × 10^6^, shown in Figure 5B, reproducing circles of lengths 4, 5 and 6 have also evolved by deletion. The length distribution converges to a state in which the 4-mers are most frequent, together with multiples of the 4-mer created by the doubling mechanism, and small numbers of 5, 6, and 7-mers which are replenished by the insertions occurring in the 4-mer. In the case where $u_{indel}$ *<< u*, the 5, 6 and 7-mers will be much less frequent than the 4-mers because selection favours rapid replication. In this case, the length distribution will consist of the minimal-length 4-mer and its multiples that arise via the doubling mechanism, as was already shown in Figure 3B.

Although we do not know with any certainty the rates of the mutation and selection processes that would have applied in the RNA World, we expect that point mutation rates associated with non-enzymatic replication would be quite large, as measure experimentally [7,8]. On the other hand, indel rates might be much smaller than this. We expect selection for increased replication rate to be strong, and therefore to operate on a short time scale of a few cell divisions. Under the assumption that insertions and deletions were rare and occurred with roughly equal frequency, then rare deletions would spread rapidly due to selection, whereas rare insertions would be eliminated by selection as fast as they could originate. This would result in a population dominated by minimal-length circles, which would have no additional non-coding regions that might evolve to encode beneficial genes. In order for a substantial numbers of longer sequences to be maintained in the population (with space available for beneficial genes), there must be a mechanism of lengthening circles that operates sufficiently rapidly to counter selection for rapidly replicating, minimal-length circles. The doubling mechanism that we have seen here, does indeed work rapidly, because it occurs on the time scale of single point mutations that render the HHRz non-functional. Thus, we argue that the doubling mechanism is important for creating a population containing circles substantially longer than the minimum length.

### 3.3. Beneficial Genes

Up to this point, we have considered circles that replicate but encode no additional function. We now introduce a ribozyme with beneficial function for the cell, represented by character **B**. Copying **B** gives its complement **b**, and vice versa. Mutations may occur with probability *u* to give a non-functional character *. We assume the **B** ribozyme is functional only when it is in a folded linear strand. It is not functional when it is part of a circular strand being used as a template, or part of the complementary strand that is being synthesized on a rolling circle. The **b** character is also assumed to be non-functional. The simulation keeps track of the number of functional ribozymes $n_{B}\;$ in each cell (i.e., the number of occurrences of the **B** character in folded linear sequences). Each functional ribozyme gives an increase β  in the rates of polymerization. For a cell with $n_{B}$ ribozymes, the rate of strand-displacement is increased to $R_{dis}\left(1+\beta n_{B}\right)$. We are assuming that beneficial ribozyme speeds up polymerization by contributing in some way to the metabolic reactions in the cell. Replication of all strands in the cell is benefitted equally, not just the strand on which the **B** gene is present. The **B** ribozyme does not represent a polymerase that binds to one specific template at a time, and it does not require to bind to a template in order to give the beneficial effect.

With the rules of the model as specified above, when a new linear strand is cleaved from a rolling circle, it folds at a rate $R_{fold}$. Cleavage can only occur between the **A** and **Z** characters. Therefore, every sequence ends with **A**. If the strand begins with a **Z** character (arising from the previous cleavage or from starting synthesis with this character), then the strand can circularize. We have assumed that folding of the sequence represents formation of the structure that brings to two ends together and allows circularization. A strand beginning with **Z** always forms a circle. A fragmentary strand not beginning with **Z** cannot form a circle and always forms a folded linear strand. When **B** genes are not present in the model, a folded linear strand plays no role, but when **B** genes are included, a folded linear strand becomes a beneficial ribozyme whenever it contains a **B** character. Thus with these rules of the model, the **B** gene can only have a useful function if it arises in a fragmentary sequence produced from the first incomplete cycle of a rolling circle. **B** genes in complete copies of the circle, always end up as new circular templates without contributing to the function of the cell.

This suggests that we need a new kind of hammerhead ribozyme whose rate of circularization is tunable. We represent the tunable ribozyme by the characters **AY**, and their complement **ya**. Whenever **AY** appears in the tail of a rolling circle, cleavage of the strand is assumed to be immediate (as with **AZ**). This produces an unfolded linear strand. Folding of all linear strands occurs at the same rate $R_{fold}$. If the strand begins with **Y**, then when folding occurs, a circle is produced with probability $f_{circ}$ and a folded linear strand with probability $1-f_{circ}$. Strands beginning with **Z** always form a circle ($f_{circ}$ = 1). Fragmentary strands that begin with neither **Y** nor **Z** always form a folded linear strand ($f_{circ}=0)$.

Adding the $f_{circ}$ parameter introduces a trade-off between circularization, which produces a new template, and folding to a linear strand, which allows the expression of a beneficial ribozyme. A wide variety of hammerhead ribozymes are known with various distinct configurations, sequences, and rates [26]. Metallic ion concentration has also been suggested to change catalytic activity in the HHRz [25]. Thus it is likely that such tunability is evolutionarily possible. As a minimal-length 4-mer without **B** genes will always benefit from forming a circle, there should be strong selection for rapidly circularizing ribozymes on minimal circles. Therefore, we began with **Z**-type ribozymes (with $f_{circ}=1$) on the minimal circles. **Y**-type ribozymes (with $f_{circ}<1$) can only be beneficial when **B** genes also exist.

We now wish to determine the range of parameters for which the presence of a **B** gene increases the reproduction rate of the cells which contain it. The most common type of sequence arising, other than the minimal 4-mer is the double-length 8-mer. Therefore, we suppose that the **B** gene arises initially on an 8-mer with sequence YzaB***A, which we call a plus strand because it encodes a functional **B** ribozyme. The complementary minus strand (accounting for circularity) is Zya***bA. The minus strand always benefits from circularization; therefore, we assume a **Z**-type ribozyme on the minus strand. The plus strand has a trade-off between circularization and expression of the **B** genes; therefore, we assume a **Y**-type ribozyme on the plus strand.

**Figure 6:**
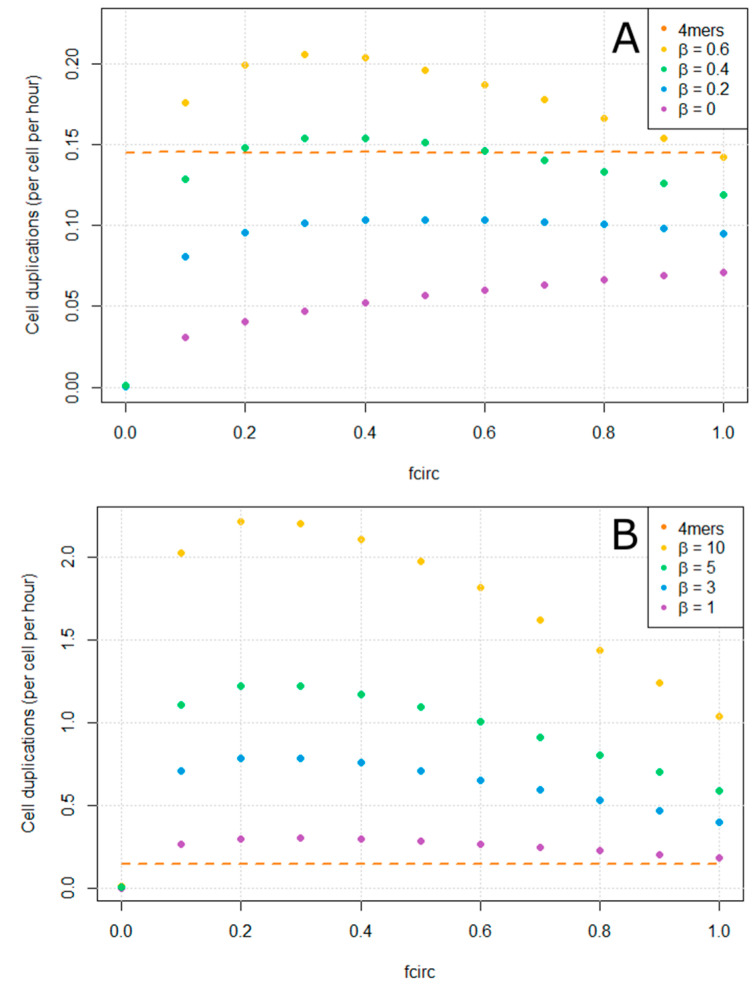
Comparison of reproductive rates of cells containing 8-mers with a beneficial gene and cells containing minimal-length 4-mers. The reproduction rate of the 8-mer cells depends on the size of the beneficial effect, *β*, and the circularization probability of the hammerhead, $f_{circ}$. There is no mutation in this figure: *u* = 0 and *u_indel_* = 0. (**A**) shows lower values of *β*, where the reproduction rate of the 8-mer cells is comparable to that of the 4-mer cells, or less. (**B**) shows higher values of *β*, where the reproduction rate of the 8-mer cells is much higher than the 4-mer cells.

We ran simulations in which each cell began with one copy of the 4-mer plus strand. After reaching a steady state, the simulation was run for a period of 10^6^ simulation steps and the average number of divisions per cell per unit time was measured. This is shown in Figure 6. For comparison, we also measure the division rate of cells that begin with the minimal 4-mer **ZzzA** (shown as a dashed line in Figure 6). The mutation and indel probabilities *u* and *u_indel_* were set to zero in these simulations, so the only replicating sequences that arise are the 8-mers or 4-mers that we begin with, and there is only one type of replicating sequence in each cell.

When there is no benefit of the **B** gene (β=0 in Figure 6A), cells containing the 8-mers always reproduce more slowly than cells containing the 4-mers. In this case, the division rate of the 8-mer cells is maximum when $f_{circ}$ is 1. There is no advantage to *not* forming a circle if the **B** gene does not produce a benefit to the cell. When β>0, there is an optimal value of $f_{circ}$ in the range 0.3 to 0.5 for the parameters shown. The trade-off favors increasing the expression of the **B** gene at the expense of reducing the number of templates. For β=0.2, the division rate of the 8-mer cells remains lower than the 4-mer cells across the whole range of $f_{circ}$. For β=0.4, the division rate of the 8-cells just exceeds the 4-mer cells when $f_{circ}$ is close to its optimal value. For β≥0.6, the division rate of the 8-cells exceeds the 4-mer cells across most of the range of $f_{circ}$. Higher values of *β* are shown in Figure 6B. For *β* > 1, the 8-mer cells reproduce much faster than the 4-mer cells. It can also be seen that even when $f_{circ}$= 1 (when a **Y** gene always circularizes), the division rate of the 8-mer cells still increases with β. So there is some benefit given by the **B** genes even when the only functional **B** ribozymes are on the incomplete fragments.

### 3.4. Spread of a Beneficial Gene

So far, we have shown that cells containing double-length circles with a beneficial genes can sometimes out-compete cells containing minimal-length circles. However, we assumed above that the longer circle with the beneficial gene was already established in a separate cell from the cells containing minimal 4-mers. More realistically, the beneficial gene is likely to first arise as a single copy inside a cell that also contains minimal 4-mers. The longer circle is at a disadvantage relative to minimal 4-mers in the same cell because it replicates more slowly. Spread of the beneficial 8-mer requires the selection at the cell level to exceed the disadvantage at the molecular level. 

In this section, we begin with a population of cells containing minimal 4-mers and allow it to come to an equilibrium under mutation *u* = 0.15, so that the cells also contain 8-mers, 12-mers, etc. We then change one * character on a single 8-mer to a **B**. We choose the 8-mer because it is the most abundant strand greater than 4. We also change the **Z** character on this same strand to a **Y** with $f_{circ}$ = 0.3, because this is close to the value at which the 8-mer cells reproduce most rapidly in Figure 6.

After introduction of the single **B** gene, we track how many cells contain circles with the **B** gene or its complement **b**. Cells can be divided into four types: those with the beneficial genes, **B** or **b**, and no 4-mers, those with 4-mers and no beneficial gene, those with both, and those with neither. 8-mer circles without **B** or **b** will arise from duplication of the 4-mers or mutation of **B** and **b** characters to *. These circles are counted as having neither a 4-mer or a beneficial 8-mer. Multiples of the 8-mer which have a B gene will arise by duplication of the 8-mers, and strands shorter than 8 with the benefit can arise from deletions. These are counted as having a benefit but no 4-mers.

**Figure 7:**
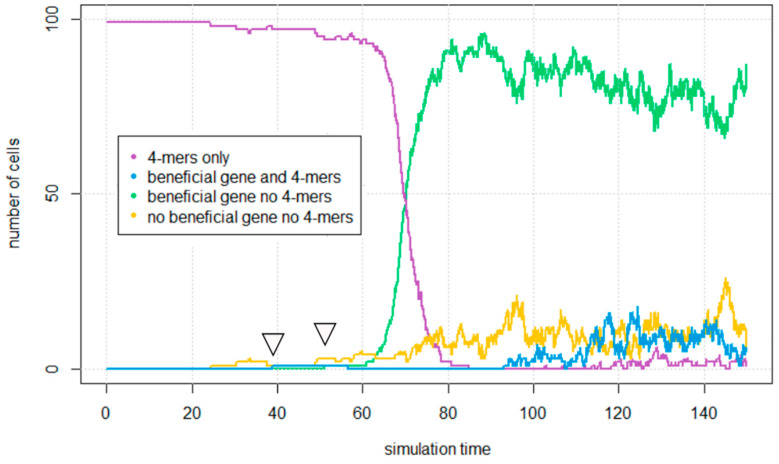
Graph showing number of cells in a 100 cell population divided into those with beneficial genes and no 4-mers, those with 4-mers and no beneficial gene, those with both, and those with neither. Time is in simulation hours. The first arrow shows the point at which the beneficial gene was added with *β* = 5. The second arrow shows the point at which the first cell appears containing beneficial 8-mers but no 4-mers. *u* = 0.15 and $u_{indel}$ = 0.015 in this graph.

The results of such a simulation are shown in Figure 7. The first arrow (at time close to 40 h) shows the point at which the single **B** gene was added with *β* = 5. Cells which contain both 4-mers and beneficial 8-mers remain rare for a long time after this. The second arrow (at time close to 50 h) shows the point at which the first cell containing beneficial 8-mers and no 4-mers appears. The appearance of the first cell of this type requires a cell-division event in which all beneficial 8-mers from a mixed cell segregate to one daughter cell while all 4-mers segregate to the other daughter. This is relatively rare, but once cells of this type are created, they rapidly multiply. In Figure 7, cells containing only beneficial 8-mers become the dominant type by about time 80 h. Mixed cells disappear at around time 80 h because they are out-competed by the cells with only beneficial 8-mers. However, this simulation also includes indels; therefore 4-mers can also be created by deletions occurring in 8-mers. This recreates mixed cells later in the simulation (around time 100 h), however these mixed cells do not take over the population, because selection against them is quite strong. 

The scenario seen in Figure 7 shows how a beneficial gene arising on a longer sequence can eventually spread to a high frequency in the population. This requires the creation of a cell that contains only beneficial 8-mer circles without any minimal circles, which is relatively rare. It is more likely that the beneficial 8-mers will disappear whilst they are still rare in the population, either due to deleterious mutations in the **B** gene or due to death of the mixed cells containing the **B** genes. If the beneficial 8-mers disappear before the creation of a cell containing only beneficial 8-mers, then they will not spread through the population. 

**Figure 8:**
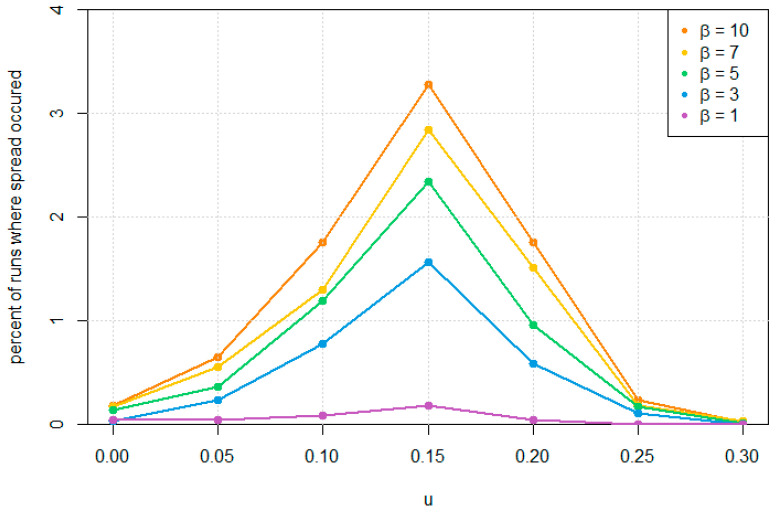
Percentage of (2 × 10^4^) runs that result in takeover by the beneficial gene in a population of 100 cells. *u* = 0.15 and *u_indel_* = 0 for all runs.

We measured how often this occurs by running our simulation multiple times. In each run, a single **B** gene was introduced on an 8-mer. The simulation was continued until one of two stop criteria was reached: either (i) the beneficial gene disappeared completely; or (ii) the number cells containing beneficial 8-mers and no 4-mers reached at least 90% of the population. The percentage of runs in which the beneficial 8-mer cells spread to high frequency is plotted against mutation probability *u* in Figure 8 for different values of the benefit parameter *β*. 

Only a small percentage of the runs lead to spread of the beneficial gene, and high values of *β* are required in order to get appreciable probabilities of spread. For *β* = 5, the maximum probability of spread is only about 2.3%. In comparison with Figure 6B, we see that the reproduction rate of 8-mer cells with *β* = 5 and $f_{circ}$ = 0.3 is approximately 8 times higher than for a 4-mer cell. A beneficial gene that gave an immediate 8-fold increase in fitness would have a very high probability of fixation in the usual approximation for the fixation rate used in population genetics [43]. However, the usual theory for the fixation probability does not apply in our case, because there are multiple strands in each cell. The reproduction rate of a cell depends on the mixture of genetic strands that it contains and also on the number of copies of folded ribozymes, which is variable. Mixed cells containing both 4-mers and 8-mers with the **B** gene do not spread to high frequencies. The spread of the **B** gene only occurs if a cell is established that contains the beneficial gene but no 4-mers (as shown in Figure 7). For this reason, the probability of spread is quite small, even when the benefit is large. To check that the **B** gene cannot spread due to spread of mixed cells, we ran additional simulations in which the run was stopped on three separate conditions: either (i) the beneficial gene dies out; (ii) cells containing the **B** gene and no 4-mers reach 90% of total; or (iii) mixed cells with both the **B** gene and 4-mers reach 90%. It was found that the runs never stopped due to criterion (iii). Thus, it was never observed that mixed cells reach high proportion.

For each value of *β* in Figure 8, a peak occurs in the probability of spread at around *u* = 0.15. The peak occurs because when *u* is very low, the 4-mers replicate accurately. There are few 8-mer (or longer) circles created. If a **B** gene arises on an 8-mer, it is in a cell that contains almost entirely 4-mers. It therefore has a low chance of spread. As *u* increases, replication of the 4-mers is less accurate, and most of the cells also contain significant numbers of 8-mers and longer circles. If a **B** gene arises on an 8-mer in this case, it has fewer 4-mers to compete with, and it is less likely to die out before the creation of a cell that contains only beneficial 8-mers. Thus the spread probability of the **B** gene is larger. If *u* is too high, however, **B** genes disappear due to deleterious mutations. We assumed that the mutation probability *u* of **B** to * is the same as that of **A** and **Z**. 

**Figure 9:**
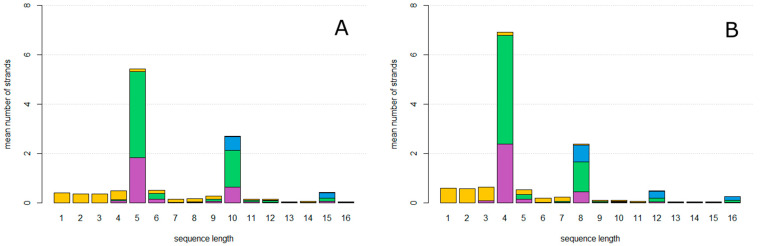
Steady state distribution of lengths beginning from 8-mer sequences YzaB***A, with *u_indel_* = 0.015 and *u* = 0.15. (**A**), *β* = 5. (**B**), *β* = 1. Colours indicate reproductive strands (green), non-cleaving strands (magenta), halving strands (blue), and non-circular fragments (yellow).

The results in Figure 7 and Figure 8 have zero indel rate, $u_{indel}$ = 0. We supposed that the **B** gene arises on an 8-mer because 8-mers are the most common type of longer sequence. However, if $u_{indel}$ is not zero, then shorter circles containing the beneficial gene can also arise by deletion. If the initial beneficial 8-mer is YzaB***A, as before, then deletions of the * characters can occur, giving 7-mers and 6-mers and, eventually, the 5-mer **YzaBA**. The **B** gene can also be deleted, giving the original minimal 4-mer **YzaA**. Thus, the long-term survival of the **B** gene depends on competition of the 5-mer and the 4-mer. We investigated this case by beginning with a population of cells each containing the 8-mer YzaB***A, and allowing the simulation to reach a steady state. When the indel rate is small ($u_{indel}$ = 0.015) and the benefit is fairly large (*β* = 5), 5-mers **YzaBA** become dominant, alongside multiples of the 5-mer which also contain **B** genes (shown in Figure 9A). If the indel rate is too large, however, or if the benefit is too small, the minimal 4-mers arise. Figure 9B shows the final steady-state distribution when *u_indel_* = 0.015 and *β* = 1. The **B** gene has been lost, and we have a distribution of the 4-mer and its multiples. 

In summary—in order for the **B** gene to survive, it has to maintain itself against deleterious mutations, deletions and selection favoring shorter sequences. These results show that this is fairly difficult, and it by no means occurs every time. However, at least sometimes, a beneficial gene becomes established. Thus, there is a route that leads from minimal, non-functional replicators towards replicating strands that encode beneficial functions.

## 4. Discussion

We have shown that the rolling circle mechanism is a feasible way of maintaining replication of RNA strands in a population of protocells. Rolling circles have the unusual property that point mutations that prevent cleavage of the HHRzs give rise to doublings of lengths of the circles, and yet the ribozyme is not eliminated by the mutation because there is a second chance of correctly copying the same template on the next passage of the circle. Under the assumption that these point mutations are more frequent than deletions that would eliminate non-essential parts of the circles, then we expect a broad distribution of sequence lengths to arise, whereas in absence of this doubling mechanism, we would expect small deletions plus selection for rapid replication to lead to almost entirely minimal length circles. We have assumed that beneficial genes can occasionally arise in non-coding regions of circles. However, the de novo appearance of beneficial genes is presumably very rare, so a mechanism is required that maintains appreciable frequencies of circles that are significantly longer than the minimal length.

As argued in the introduction, our principal reason for considering circular templates is that we require a strand displacement mechanism to avoid product inhibition and this is only likely to work on a circular template. However, this leads to several other points that are relevant for the evolution of chromosomes carrying useful genes. We assumed that the first circuit of the rolling circle occurs at a more rapid rate than subsequent circuits because the first one does not require strand displacement, whereas subsequent circuits do. For this reason, a circular template is almost always in a double-stranded state. This has important consequences for the stability of the genetic molecule, because it is known that double stranded RNAs are much less prone to degradation than single strands. Another advantage of circular strands as templates might be increased processivity of polymerase ribozymes. Recently developed polymerases have a clamp domain that wraps around the template [6]; hence if the template is circular, the polymerase can proceed multiple times around the same template.

In RNA World models there is always an apparent conflict between the need of a sequence to act both as a gene and a ribozyme. Presumably the folding of a strand to a functional ribozyme structure prevents its operation as a template. The rolling circle mechanism leads to an immediate distinction between double-stranded circles that are used as templates and folded linear single strands that function as ribozymes. We have pointed out that the relative rate of formation of folded strands to new circular templates is a tunable, evolvable property of the HHRzs (modelled by the $f_{circ}$ parameter). If the circle does not encode beneficial genes, then it should always re-circularize as fast as possible ($f_{circ}=1)$. The same is true for the negative strand of a circle encoding a beneficial ribozyme. However, the positive strand requires a balance between folding and re-circularizing, meaning that there is an optimal value of $f_{circ}$, as shown in Figure 6. The independent evolution of $f_{circ}$ on plus and minus strands allows the relative numbers of plus and minus circles and folded ribozymes to be optimized to increase overall replication rate. The division of labour between template and catalyst has been discussed previously in the context of the origin of DNA [44], but in the current model this arises naturally in the RNA World without the need for a second kind of genetic polymer.

In the current paper, we have only considered circles with a single beneficial gene, but rolling circles could in principle encode multiple types of beneficial genes, as proposed in [33]. Placing multiple genes on a chromosome strand is beneficial from the point of view of coordinating gene replication, but it introduces the need for a mechanism to allow folding of functional ribozymes on separate linear strands—i.e., a need to distinguish transcription of a single ribozyme from replication of a chromosome. In the rolling circle mechanism, different beneficial genes on the same strand could be separated by copies of HHRzs, which would allow some strands to be cleaved into separate single folded ribozymes while other strands re-circularize and become templates. This might avoid the need to evolve separate transcriptional start and stop signals for each gene. We suspect that this would only work for a relatively small number of genes, however, because it will be necessary at least sometimes to complete replication of the whole circle before cleavage occurs at intermediate positions. This problem would increase the advantage of separate smaller circles encoding a single gene relative to longer circles with multiple genes.

In a single cell, shorter circles always replicate faster than longer ones. When considering the origins of the 8-mer circles containing the beneficial gene in Figure 7, we showed that the longer circle only spreads after it eventually manages to get into a cell that does not contain any 4-mers. A similar issue would arise when considering competition between separate circles containing one beneficial gene each and longer circles with multiple genes. However, once a multi-gene circle becomes established in a separate cell, this cell is likely to multiply rapidly.

In summary, the occurrence of circles in abiotic RNA polymerization [21,22], the ease with which self-cleaving ribozymes arise de novo [30], the ability of polymerase ribozymes to copy circular templates [6,19], and the natural occurrence of circular viroids [18] all point to the importance of circular templates in RNA replication. Developments in experimental methods for non-enzymatic and ribozyme-catalyzed replication may soon make it possible to study the evolution and replication of circular templates in experiments and potentially advance our understanding of the origins of life.

## Figures and Tables

**Table 1:** Example of a series of events occurring on a circular template. Sequences before the colon are template circles. Sequences after the colon are complementary strands. Underlined sequences in the complement are single-stranded tails.

Event	From State	*Last*	To State	*Last*	Rate
1	**ZzaA**	-	**ZzaA: A**	3	$R_{syn}$
2	**ZzaA: A**	3	**ZzaA: AZ**	2	$R_{syn}$
3	**ZzaA: AZ**	2	**ZzaA: AZz**	1	$R_{syn}$
4	**ZzaA: AZz**	1	**ZzaA: AZza**	4	$R_{syn}$
5	**ZzaA: AZza**	4	**ZzaA:** **A** **ZzaA**	3	$R_{dis}$
6 cleavage	**ZzaA: AZzaA**	3	**ZzaA:** **AZ** **zaAZ** **→ A + ZzaA:** **Z** **zaAZ**	2 2	$R_{dis}$
7 cleavage	**ZzaA:** **ZzaA** **ZzaA**	3	**ZzaA:** **ZzaAZ** **zaAZ** **→ ZzaA + ZzaA:** **Z** **zaAZ**	2 2	$R_{dis}$

**Table 2:** Standard values for model parameters.

Reaction	Rate	Description
$R_{dis}$	1 h^−1^	Rate of copying a single character when strand displacement is required
$R_{syn}$	100 h^−1^	Rate of copying a single character when synthesis is possible without strand displacement
$R_{fold}$	100 h^−1^	Rate at which a linear strand folds into its secondary structure
$R_{loss}$	1× 10^−3^ h^−1^	Rate at which a cell dies from lack of materials
N	200	Number of protocells in population
$S_{o}$	20	Maximum number of strands per protocell

## References

[1] Robertson M.P., Joyce G.F. (2012). The origins of the RNA world. Cold Spring Harb. Perspect. Biol..

[2] Higgs P.G., Lehman N. (2015). The RNA World: Molecular cooperation at the origins of life. Nat. Rev. Genet..

[3] Attwater J., Wochner A., Holliger P. (2013). In-ice evolution of RNA polymerase ribozyme activity. Nat. Chem..

[4] Horning D.P., Joyce G.F. (2016). Amplification of RNA by an RNA polymerase ribozyme. Proc. Natl. Acad. Sci. USA.

[5] Attwater J., Raguram A., Morgunov A.S., Gianni E., Holliger P. (2018). Ribozyme-catalysed RNA synthesis using triplet building blocks. eLife.

[6] Cojocaru R., Unrau P.L. (2021). Processive RNA polymerization and promoter recognition in an RNA World. Science.

[7] Leu K., Obermayer B., Rajamani S., Gerland U., Chen I.A. (2011). The prebiotic evolutionary advantage of transferring genetic information from RNA to DNA. Nucleic Acids Res..

[8] Bapat N.V., Rajamani S. (2015). Effect of Co-solutes on Template-Directed Nonenzymatic Replication of Nucleic Acids. J. Mol. Evol..

[9] Prywes N., Blain J.C., Del Frate F., Szostak J.W. (2016). Nonenzymatic copying of RNA templates containing all four letters is catalyzed by activated oligonucleotides. eLife.

[10] O’Flaherty D.K., Kamat N.P., Mirza F.N., Li L., Prywes N., Szostak J.W. (2018). Copying of Mixed-Sequence RNA Templates inside Model Protocells. J. Am. Chem. Soc..

[11] Sosson M., Richert C. (2018). Enzyme-free genetic copying of DNA and RNA sequences. Beilstein J. Org. Chem..

[12] Sosson M., Pfeffer D., Richert C. (2019). Enzyme-free ligation of dimers and trimers to RNA primers. Nucleic Acids Res..

[13] Tupper A.S., Higgs P.G. (2021). Rolling-circle and strand-displacement mechanisms for non-enzymatic RNA replication at the time of the origin of life. J. Theor. Biol..

[14] Zhou L., Ding D., Szostak J.W. (2021). The Virtual Circular Genome Model for Primordial RNA Replication. RNA.

[15] Wachowius F., Holliger P. (2019). Non-Enzymatic Assembly of a Minimized RNA Polymerase Ribozyme. Chem. Syst. Chem..

[16] Zhou L., O’Flaherty D.K., Szostak J.W. (2020). Assembly of a Ribozyme Ligase from Short Oligomers by Nonenzymatic Ligation. J. Am. Chem Soc..

[17] Chamanian P., Higgs P.G. (2022). Computer simulations of template-directed RNA synthesis driven by temperature cycling in diverse sequence mixtures. PLoS Comp. Biol..

[18] Flores R., Gago-Zachert S., Serra P., Sanjuan R., Elena S.F. (2014). Viroids: Survivors from the RNA world?. Annu. Rev. Microbiol..

[19] Diener T.O. (1989). Circular RNAs: Relics of precellular evolution?. Proc. Nat. Acad. Sci. USA.

[20] Diener T.O. (2016). Viroids: “living fossils” of primordial RNAs?. Biol. Direct..

[21] Kristoffersen E.L., Burman M., Noy A., Holliger P. (2022). Rolling circle RNA synthesis catalyzed by RNA. eLife.

[22] Zhou L., Kim S.C., Ho K.H., O’Flaherty D.K., Giurgiu C., Wright T.H., Szostak J.W. (2019). Non-enzymatic primer extension with strand displacement. eLife.

[23] Hassenkam T., Damer B., Mednick G., Deamer D. (2020). AFM images of viroid-size rings thatself-assemble from mononucleotides through wet-dry cycling: Implications for the origin of life. Life.

[24] Hassenkam T., Deamer D. (2022). Visualizing RNA polymers produced by hot wet-dry cycling. Sci. Rep..

[25] Altman S., Baer M., Guerrier-Takada C., Vioque A. (1986). Enzymatic cleavage of RNA by RNA. Trends Biochem. Sci..

[26] Cech T.R. (1987). The Chemistry of Self-Splicing RNA and RNA Enzymes. Science.

[27] Conaty J., Hendry P., Lockett T. (1999). Selected classes of minimised hammerhead ribozyme have very high cleavage rates at low Mg^2+^ concentration. Nucleic Acids Res..

[28] Boots J.L., Canny M.D., Azimi E., Pardi A. (2008). Metal ion specificities for folding and cleavage activity in the Schistosoma hammerhead ribozyme. RNA.

[29] Ferré-D’Amaré A.R., Scott W.G. (2010). Small Self-cleaving Ribozymes. Cold Spring Harb. Perspect. Biol..

[30] Hammann C., Luptak A., Perreault J., de la Peña M. (2012). The ubiquitous hammerhead ribozyme. RNA.

[31] O’Rourke S.M., Scott W.G. (2018). Structural Simplicity and Mechanistic Complexity in the Hammerhead Ribozyme. Prog. Mol. Biol. Transl. Sci..

[32] Salehi-Ashtiani K., Szostak J.W. (2001). In vitro evolution suggests multiple origins for the hammerhead ribozyme. Nature.

[33] Bianconi G., Zhao K., Chen I.A., Nowak M.A. (2013). Selection for Replicases in Protocells. PLoS Comput. Biol..

[34] Ma W., Hu J. (2012). Computer Simulation on the Cooperation of Functional Molecules during the Early Stages of Evolution. PLoS ONE.

[35] Ma W., Yu C., Zhang W. (2013). Circularity and self-cleavage as a strategy for the emergence of a chromosome in the RNA-based protocell. Biol. Direct..

[36] Kim Y.E., Higgs P.G. (2016). Co-operation Between Polymerases and Nucleotide Synthetases in the RNA World. PLoS Comput. Biol..

[37] Roy S., Sengupta S. (2021). Evolution towards increasing complexity through functional diversification in a protocell model of the RNA World. Proc. R. Soc. B.

[38] Maynard Smith J., Szathmary E. (1993). The origin of chromosomes I. Selection for linkage. J. Biol..

[39] Gillespie D.T. (1977). Exact stochastic simulation of coupled chemical reactions. J. Phys. Chem..

[40] Shah V., de Bouter J., Pauli Q., Tupper A.S., Higgs P.G. (2019). Survival of RNA replicators is much easier in protocells than in surface-based, spatial systems. Life.

[41] Macey J.R., Larson A., Ananjeva N.B., Papenfuss T.J. (1997). Replication slippage may cause parallel evolution in secondary structures of mitochondrial transfer RNAs. Mol. Biol. Evol..

[42] Hancock J.M., Chaleeprom W., Dale J., Gibbs A. (1995). Replication slippage in the evolution of potyviruses. J. Gen. Virol..

[43] Kimura M. (1962). On the probability of fixation of mutant genes in a population. Genetics.

[44] Takeuchi N., Hogeweg P., Koonin E.V. (2011). The origin of DNA genomes: Evolution of the division of labour between template and catalyst in model replicator systems. PLoS Comp. Biol..

